# Implementation of a Best Practice Advisory to alert inpatient providers of necessary discharge prescriptions for insulin and supplies for patients with diabetes

**DOI:** 10.1016/j.jcte.2025.100428

**Published:** 2025-12-22

**Authors:** Michelle D. Lundholm, Allison Weathers, Shannon Knapp, Pratibha P.R. Rao

**Affiliations:** a Department of Endocrinology, Medical Specialty Institute, Cleveland Clinic, Cleveland, OH, USA; b Information Technology Division (ALW), Cleveland Clinic, Cleveland, OH, USA

**Keywords:** Diabetes, Hospital discharge, Quality improvement, Patient safety, Transition of care, Best practice advisory

## Abstract

**Background:**

Medication discrepancies and omissions at hospital discharge are a common, preventable source of harm for patients with diabetes. Ensuring accurate prescriptions for insulin and diabetes supplies is critical for safe transitions of care. We aimed to evaluate the impact of a Best Practice Advisory (BPA) integrated into the electronic medical record (EMR) on the accuracy of diabetes-related discharge prescriptions.

**Methods:**

This quality improvement initiative was implemented across seven hospitals within a large health system. The BPA was triggered in the EMR discharge navigator for patients evaluated by a Diabetes Care and Education Specialist (DCES), prompting providers to review and order recommended diabetes medications and supplies. BPA utilization data were collected from February to December 2022. A retrospective chart review of 100 consecutive eligible patients discharged in December 2022 seen by DCES was assessed for prescription accuracy and supply provision.

**Results:**

The BPA was triggered 6,714 times for 2,266 patient encounters over 11 months. Among 100 reviewed patients (mean age 57 years, 58 % male, 76 % type 2 diabetes), insulin prescription accuracy at discharge was 99 %, and 88 % received all recommended diabetes supplies. Only 1 % of patients contacted the hospital post discharge for additional supplies, suggesting most omissions were clinically appropriate.

**Conclusions:**

Integration of a BPA into the EMR significantly improved the accuracy and completeness of diabetes-related discharge prescriptions. This low-cost, sustainable digital intervention enhances patient safety and care quality, and represents a scalable model for improving discharge practices in inpatient settings.

## Highlights

• An EMR Best Practice Advisory enhanced diabetes prescription accuracy on discharge.
• 99% of patients got correct insulin type and formulation (pen or vial) of insulin.
• 88% of patients received all recommended diabetes supplies at discharge.
• Digital intervention is low-cost, sustainable, and scalable for diabetes care.

## Summary

This quality improvement study implemented a Best Practice Advisory in the electronic medical record to enhance diabetes-related discharge prescriptions. The intervention promoted high insulin prescription accuracy (99%) and diabetes supply provision (88%). Results demonstrate the effectiveness of targeted digital interventions in improving diabetes care quality at hospital discharge.

## Introduction

Medication discrepancies and prescription omissions at hospital discharge represent a common, preventable source of potential patient harm and adverse health outcomes [1], [2]. These prescribing errors are frequently observed with diabetes medications. At baseline, 30 % of patients newly initiated on insulin therapy at our institution did not receive appropriate diabetes-related prescriptions upon hospital discharge in 2019 [3]. Inappropriate discharge prescriptions include missing or incorrect delivery devices or supplies, which may prevent patients from following discharge instructions and compromise medication safety after discharge. The growing prevalence of diabetes, affecting 11 % of the US population, underscores the ongoing challenge of ensuring safe and comprehensive discharge practices for patients with both new-onset and pre-existing diabetes [4], [5], [6]. Structured interventions such as discharge medication reconciliation and coordinated follow-up have been demonstrated to reduce medication-related problems in this population [7].

In keeping with current diabetes guidelines, inpatient providers at our institution typically consult an inpatient Diabetes Care and Education Specialist (DCES) for education on diabetes management, especially when a patient will be discharged on a new diabetes treatment regimen [8], [9]. Our initiative aims to further enhance the insulin and diabetes-supply prescription accuracy for all patients evaluated by a DCES. This is achieved by integrating a Best Practice Advisory (BPA) within our electronic medical record (EMR) designed to alert discharging providers to specific diabetes management recommendations at discharge.

## Methods

**Fig. 1:**
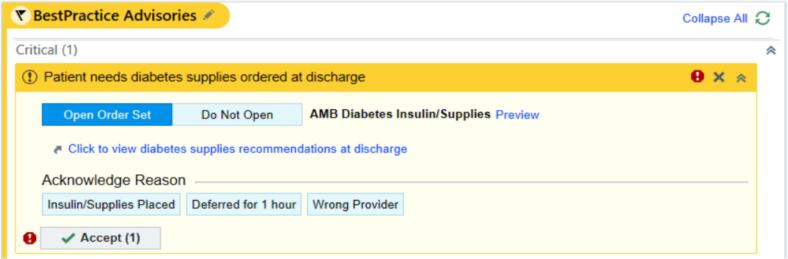
This project’s Best Practice Advisory alert as it appears in the Epic discharge navigator for any patient that has been seen by a Diabetes Care and Education Specialist during their admission. It contains links to open diabetes insulin/supplies order set and to view the recommendations.

In this quality improvement and patient safety project, we implemented an enterprise-wise BPA within the discharge navigator of our system’s EMR (Epic) in February of 2022. This BPA is triggered on opening the discharge navigator for patients who were seen by a trained DCES during their hospitalization (Fig. 1). The development of this BPA was a collaborative effort involving endocrinologists, DCES, and colleagues from the information technology (IT) division including physician clinical informaticists, with implementation support from our Epic system experts. A root cause analysis, process map, and plan-do-study-act (PDSA) cycle for this project are provided in the Supplemental material.

**Fig. 2:**
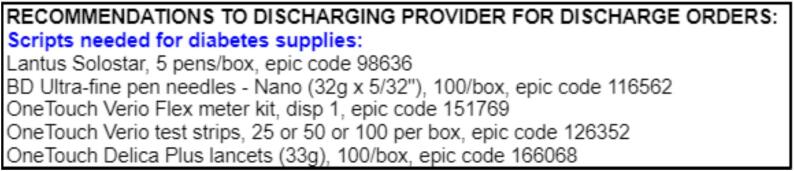
Example diabetes supplies recommendations that appear on the side panel with details including brands, amounts, and Epic codes.

The BPA is currently operational in our Main Campus and six Northeast Ohio regional hospitals where DCES staff are trained in the use of an activating “SmartPhrase” in their consultation note. Upon activation, it alerts the primary provider to DCES-recommended diabetes supplies in the discharge navigator. These supply categories are insulin, insulin delivery supplies (pen needles, syringes), glucometers, test strips, and lancets. A single click by the discharging provider opens a side panel displaying patient-specific recommendations, tailored to the individual’s insurance coverage, complete with corresponding Epic order codes (Fig. 2). Additionally, the BPA provides a direct link to the ambulatory diabetes insulin and supplies order set. To ensure appropriate action, the BPA remains active until the provider confirms that the recommended diabetes supplies have been ordered. Options are available to defer the alert for one hour or to indicate if the user is not the discharging provider.

For this quality improvement initiative, we analyzed BPA utilization data from February through December of 2022. This included all patients seen by DCES during their hospitalization in this time frame. To assess the intervention’s efficacy, we conducted a retrospective chart review of 100 consecutive patients who were seen by DCES while hospitalized in December 2022. This window, near the end of the 11-month study period, was selected to allow time for users to accommodate to the BPA. Accuracy was defined as the percentage of patients whose discharge prescriptions matched the recommendations from DCES. For insulin prescriptions, this included correct type and formulation of insulin. Data analysis was performed using descriptive statistics.

## Results

The BPA implemented in the EMR demonstrated significant utilization: over an 11-month period, the BPA was triggered 6,714 times for 3,049 DCES visits to 2,154 unique patients and 2,266 unique patient encounters across our seven hospitals. This reflects that the discharge navigator was opened on average approximately 3 times per patient hospitalization before the discharging healthcare professional confirmed that the DCES-recommended diabetes supplies had been ordered.

Of 100 patients who were sampled from consecutive hospitalizations, 58 % (N = 58) were male, 60 % (N = 60) non-Hispanic white, with a mean age of 57.0 ± 15.7 years and HgA_1c_ of 8.7 ± 2.6 % (Table 1). The distribution of diabetes types was predominantly type 2 (76 %, N = 76) with several type 1 (11 %, N = 11) or other causes (13 %, N = 13, e.g. steroid-induced or pancreatic abnormalities). Twenty-seven percent (N = 27) of these patients were newly diagnosed with diabetes during their hospitalization. As for hospitalization features, patients were hospitalized for an average of 7.6 ± 7.3 days. The primary teams were 77 % medical and 23 % surgical.

**Table 1:** Patient characteristics from 100 consecutive patients admitted in December 2022 and seen by DCES.

Feature	N (%) or Mean ± SD
Sex, male	58 (58 %)

Race	
White, non-Hispanic	60 (60 %)
White, Hispanic	5 (5 %)
Black	33 (33 %)
Multiracial	2 (2 %)
Mean age (years)	57.0 ± 15.7
Mean HgA_1c_ (%)	8.7 ± 2.6 %

Diabetes type	
Type 1	11 (11 %)
Type 2	76 (76 %)
Other	13 (13 %)
New onset diabetes	27 (27 %)

Following implementation of the BPA, there was a high accuracy of insulin and supply orders with breakdown provided in Table 2. Of insulin prescriptions, there was one patient (1 %, N = 1) who was recommended basal and prandial insulin but discharged on basal insulin and metformin instead. Overall, 99 % (N = 99) of patients received the correct type and formulation (pen or vial) of insulin as designated by DCES at discharge, or did not require such a prescription. Additionally, 88 % (N = 88) of patients received prescriptions for all DCES-recommended supplies. Specifically, insulin pen needles were prescribed in N = 49 of 54 recommended cases (90.7 %), glucometer in N = 17 of 22 cases (77.3 %), test strips in N = 73 of 78 cases (93.6 %), and lancets in N = 66 of 69 cases (95.7 %). Of those who were missing a diabetes supply, 75 % (N = 9 of 12) had a pre-existing diagnosis of diabetes. Despite the omissions, only 1 % (N = 1) contacted the hospital after discharge requesting additional supplies.

**Table 2:** Summary of DCES-recommended prescriptions and number of times the recommendation was ordered.

Prescription	Number of times DCES-recommended, N	Number of times recommendation was ordered, N (%)
Insulin		
Basal	39	39 (100 %)
Prandial	45	44 (97.8 %)
Mixed	7	7 (100 %)

Insulin supplies		
Insulin pen needles	54	49 (90.7 %)
Syringes	0	0 (N/A)

Glucose monitoring supplies		
Glucometer	22	17 (77.3 %)
Test strips	78	73 (93.6 %)
Lancets	69	66 (95.7 %)

## Discussion

This quality improvement initiative demonstrates the effectiveness of a targeted EMR intervention in enhancing the accuracy and completeness of diabetes-related discharge prescriptions. The implementation of a BPA within the discharge navigator resulted in a remarkably high rate of appropriate insulin prescriptions (99 %) and a high provision of necessary diabetes supplies (88 %) for patients seen by a DCES during their hospitalization.

The success of this intervention can be attributed to several key factors. First, the collaborative design process ensured that the BPA was tailored to clinical needs and workflow integration. Second, the BPA's user-friendly interface, which offered instant access to patient-specific recommendations and order sets, facilitated provider compliance. Third, the requirement for active acknowledgment of the BPA ensured that providers consciously addressed diabetes management needs at discharge.

It is important to note that some patients (12 %) still lacked one or more recommended supplies at discharge. However, the low rate of post-discharge supply requests (1 %) suggests that some omissions may have been intentional or clinically appropriate. This is supported by the observation that most patients with a supply omission had a pre-existing diagnosis of diabetes and may have already had sufficient supplies at home. Glucometer was the most frequently omitted prescription, which may have been intentional, as several of our hospitals routinely provide free sample glucometers that would preclude the need for a formal prescription. These findings highlight the continued importance of individualized discharge planning beyond what is provided by a BPA.

A notable strength of this intervention lies in its minimal implementation costs and high sustainability. The BPA leverages existing EMR infrastructure and the primary investment was in the design process and staff training. Moreover, the sustainability is evident in its seamless integration into existing clinical workflows to become a consistent, standard part of care delivery, which has continued beyond the study period.

Future study may involve expanding the BPA to include other types of diabetes medications besides insulin, and the potential to expand this initiative to other centers. While we have only studied its use in our Main Campus and Northeastern Ohio regional hospitals, expanding this initiative to other centers could provide insights into its generalizability and effectiveness in diverse clinical settings. Our results suggest that similar interventions could be adopted in health systems with comparable capabilities, potentially leading to improvements in diabetes care quality on a broader scale.

In conclusion, this quality improvement project demonstrates that a well-designed, EMR-integrated intervention can promote high-quality diabetes care at hospital discharge. By ensuring that patients receive appropriate medications and supplies, this approach has the potential to improve post-discharge diabetes management. As healthcare systems continue to grapple with the effective management of diabetes, such targeted digital interventions offer a promising strategy for enhancing care quality and patient safety.

## CRediT authorship contribution statement

**Michelle D. Lundholm:** Writing – review & editing, Writing – original draft, Formal analysis, Data curation. **Allison Weathers:** Writing – review & editing, Resources, Methodology, Conceptualization. **Shannon Knapp:** Writing – review & editing, Resources, Methodology, Conceptualization. **Pratibha P.R. Rao:** Writing – review & editing, Supervision, Methodology, Investigation, Data curation, Conceptualization.

## Declaration of competing interest

The authors declare that they have no known competing financial interests or personal relationships that could have appeared to influence the work reported in this paper.
