# Supplementary material for: Implementation of a Best Practice Advisory to alert inpatient providers of necessary discharge prescriptions for insulin and supplies for patients with diabetes
Source: J Clin Transl Endocrinol. 2025 Dec 22;43:100428. doi: 10.1016/j.jcte.2025.100428 (PMC12807630; doi:10.1016/j.jcte.2025.100428)

**Supplemental Material**

**Implementation of a Best Practice Advisory to Alert Inpatient Providers of Necessary Discharge Prescriptions for Insulin and Supplies for Patients with Diabetes**

**Table S1.** Root cause analysis for inaccurate diabetes discharge medications

| Root cause category | Specific root causes |
| --- | --- |
| Human factors | - Lack of experience prescribing insulin and related supplies - Lack of awareness of DCES recommendations - Manual medication reconciliation errors |
| System factors | - Fragmented communication between providers (difficulty finding relevant notes/recommendations) - Lack of easy-to-use diabetes order set |
| Environmental factors | - High workload and time constraints |

**Figure S1.** Process map


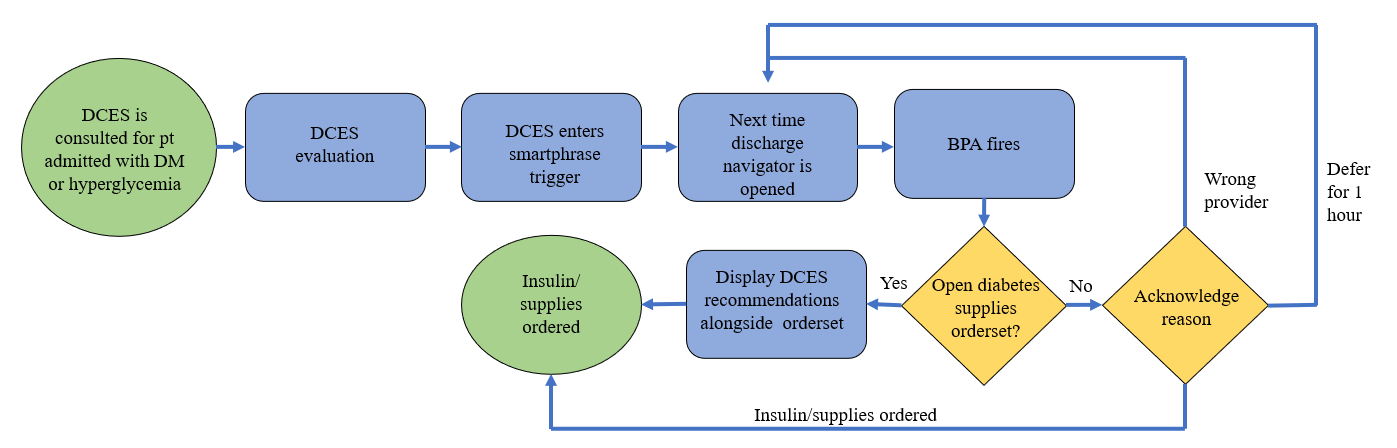


**Figure S2.** PDSA cycle reflected in this quality improvement project (one cycle)


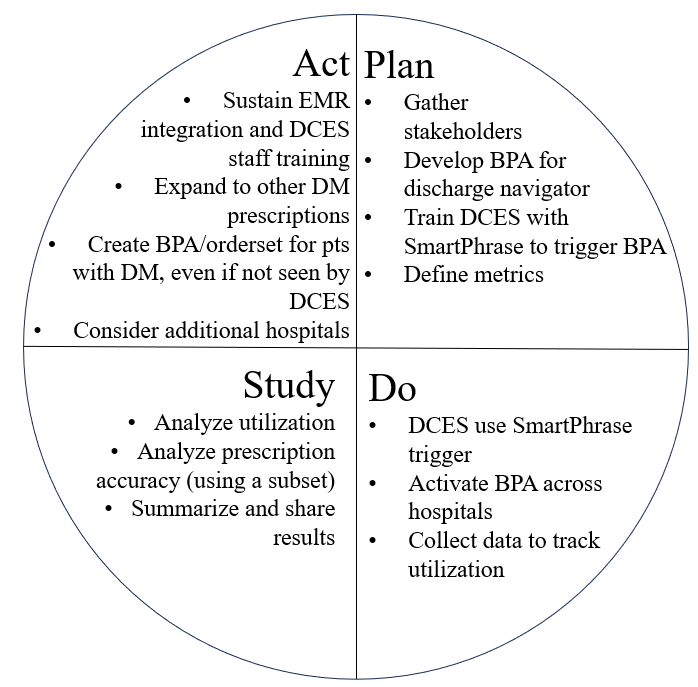

Supplement: Supplementary Data 1 [file mmc1.docx]
